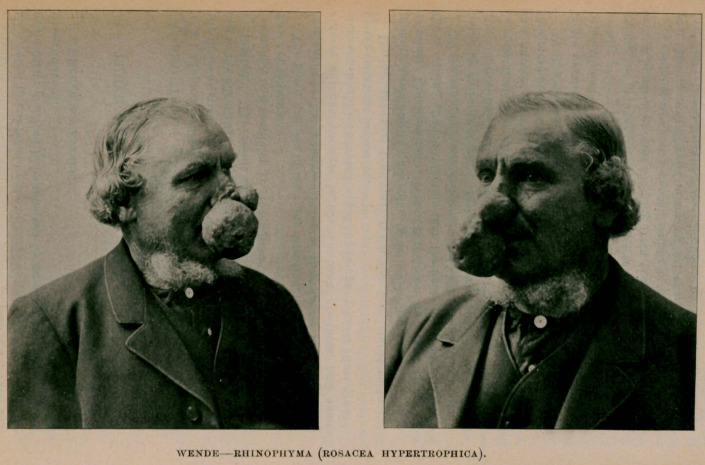# A Case of Rhinophyma (Rosacea Hypertrophica)

**Published:** 1895-08

**Authors:** Ernest Wende

**Affiliations:** Buffalo. N. Y., Clinical professor of dermatology, University of Buffalo.


### A CASE OF RHINOPHYMA (ROSACEA HYPERTROPHICA).

By ERNEST WENDE, M. D., Buffalo, N. Y.,
Clinical professor of dermatology, University of Buffalo.

THE subject of the accompanying portrait is a German, aged 63, and by occupation a dealer in pig's feet. Apart from the existence of a squamous eczema of the left leg, and his facial embellishment, he is to all appearances healthy.

He gives the following history: Twenty years ago, subsequent to an attack of typhoid, he first noticed a daily temporary flushing of the face, which phenomena continued for several months, when the hyperemia induced became permanent; and, coincident with it, there occurred appearance of numerous papules, pustules and nodules. They were well disseminated over the face and neck, the greater number, however, occurring upon the cheeks and chin.

Four years later he observed a small excrescence arising from a papule seated on the right side of the nose, near the margin, midway between the tip and ala. In its incipiency, the development was very gradual, but it finally grew rapidly to proportions of considerable dimensions, which can be best appreciated by referring to the illustrations.

Recently, it has persisted unchanged with the exception that it has become somewhat paler in color. It was ascertained, by means of a pair of calipers, that the measurement of the longest diameter, from its pendulous attachment to base, is four and three-fourths inches; transversely with the face, three and one-fourth inches, and at right angles with the latter, three inches.

In addition to the neoplasm just described, there are eight similar tumors situated about the tip of the nose, the larger of which is nearly round and measures one and one-half inches in diameter. The small ones are sessile, while the larger two have pedicles and dangle at every movement of the head. To the touch, the consistency of these growths seem doughy and elastic. Their surface is pitted with numerous pores, some of which gape widely, and upon pressure a vast quantity of sebum, having a disagreeable, rancid odor, can be liberated. At one time their color, as well as that of the nose, was of a purplish hue; it has now, with advancing age, assumed almost a normal tint. Furthermore, their surface and that of the nose and cheeks, in close proximity, is shiny and oily and traversed by dilated and tortuous blood-vessels. The nasal cartilage and mucous membrane are apparently healthy.

Barring the disfigurement, the sensation of weight, the interference in seeing the movements of the mouth, and inconveniences offered in eating and drinking, there are no disagreeable features.

In taking his beer he raises the neoplasm with one hand upward upon its elastic pedicle or hinge, and with the other the glass is put to his mouth.

Concerning the individual's habits, it may be stated that he is a moderate beer drinker; also, owing to a recent seizure of gravel, he has indulged in the morning in a drink or two of gin and juniper-berry tea.

In accordance with the views of some authorities, alcohol is closely connected as the etiological factor of a red nose and its ornaments. However, I am of the opinion that there is generally required some determining cause outside of alcoholic beverages, for tipplers are common while rhinophyma is rare—and, again, it has occurred in persons of temperate, even abstemious, habits.

WENDE—RHINOPHYMA (ROSACEA HYPERTROPHICA).

Were it not for the emphatic objections offered against any and all surgical interference by the owner, this dermatological curiosity could easily and readily be removed by excision and a presentable nose produced.

471 Delaware Avenue.

## Figures and Tables

**Figure f1:**